# Evidence‐ and consensus‐based guideline on lichen sclerosus

**DOI:** 10.1111/ddg.70000

**Published:** 2026-03-04

**Authors:** Gudula Kirtschig, Linn Woelber, Andreas Günthert, Karl Becker, Alexandra Ciresa‐König, Bettina Fischer, Susanne Fricke‐Otto, Claudia Günther, Christine Hirchenhain, Narayani Helga Köllmann, Alexander Kreuter, Bartosz Malisiewicz, Felix Neis, Dan mon O'Dey, Hagen Ott, Martin Promm, Regina Renner, Anne‐Rose Schardt, Ines Schweizer, Raimund Stein, Jan Ter‐Nedden, Gerda Trutnovsky, Agnes Wand, Gerhard Weyandt, Ricardo N. Werner, Maria Kinberger

**Affiliations:** ^1^ Medbase Health Centre Frauenfeld Frauenfeld Switzerland; ^2^ Dysplasia Center Jerusalem Hospital University Hospital Hamburg‐Eppendorf Hamburg Germany; ^3^ gyn‐zentrum Luzern Cham Kriens Lucerne Switzerland; ^4^ Office for Pediatric Surgery Bonn Bonn Germany; ^5^ University Hospital for Gynecology and Obstetrics Medical University Innsbruck Innsbruck Austria; ^6^ Swiss Lichen Sclerosus Association Dottikon Switzerland; ^7^ Department of Pediatric Endocrinology and Diabetes Pediatric Emergency Treatment HELIOS Hospital Krefeld Krefeld Germany; ^8^ Department of Dermatology University Hospital Carl Gustav Carus Dresden Dresden Germany; ^9^ Department of Gynecology and Obstetrics University Hospital Carl Gustav Carus Dresden Dresden Germany; ^10^ Department of Dermatology Venereology and Allergology Helios St. Elisabeth Hospital Oberhausen Oberhausen Germany; ^11^ Department of Dermatology Venereology and Allergology Helios St. Johannes Hospital Duisburg Duisburg Germany; ^12^ Main Medic. All Office Frankfurt am Main Germany; ^13^ Department of Gynecology and Obstetrics University Hospital Tübingen Tübingen Germany; ^14^ Luisenhospital Aachen/Department of Plastic Reconstructive and Aesthetic Surgery Hand Surgery Center for Reconstructive Surgery of Female Genitalia Aachen Germany; ^15^ MUC‐iSPZ Hauner LMU Center for Children With Medical and Developmental Complexity Ludwig Maximilian University Munich Munich Germany; ^16^ Barmherzige Brüder Regensburg Department of Pediatric Urology Regensburg Germany; ^17^ Dermatology Office Esslingen Esslingen Germany; ^18^ Joint Office Ihre Frauenärztinnen im Taunusstein Taunusstein Germany; ^19^ Office for Sexual and Psychotherapy Lucerne Switzerland; ^20^ Center for Pediatric Adolescent and Reconstructive Urology University Medical Center Mannheim Mannheim Germany; ^21^ Dermatopathological and Pathological Laboratory Center Hamburg Hamburg Germany; ^22^ Department of Gynecology and Obstetrics Medical University of Graz Graz Austria; ^23^ Alice Salomon University Berlin Berlin Germany; ^24^ Department of Dermatology Allergology and Venereology Bayreuth Hospital Bayreuth Germany; ^25^ Division of Evidence‐Based Medicine (dEBM) Department of Dermatology Venereology and Allergology Charité – Universitätsmedizin Berlin corporate member of Freie Universität Berlin and Humboldt‐Universität zu Berlin Berlin Germany

**Keywords:** Evidence‐based guideline, guideline, lichen sclerosus

## Abstract

The German‐language, consensus‐ and evidence‐based S3 guideline on lichen sclerosus (LS) was developed based on the European “EuroGuiDerm Guideline on lichen sclerosus” under the leadership of the *German Dermatological Society* (DDG) and the *German Society for Gynecology and Obstetrics* (DGGG). Particular emphasis was placed on adapting the recommendations to the healthcare conditions in German‐speaking countries. The interdisciplinary guideline development group consisted of 24 experts from 16 medical societies and actively included patient representatives in the development process.

The guideline provides comprehensive recommendations on diagnosis, patient management, follow‐up care, and patient education, as well as the treatment of both genital and extragenital LS in women, men, girls, and boys. Regardless of age or sex, ultrapotent or potent topical glucocorticosteroids in combination with emollients remain the standard therapy for genital LS. In male patients with LS‐associated phimosis who do not respond sufficiently to standard therapy, circumcision with complete removal of the foreskin is indicated. For extragenital LS, phototherapy with UV light is recommended as an adjunct to topical treatment. Topical calcineurin inhibitors are second‐line therapy.

## INTRODUCTION

This German guideline on lichen sclerosus is an adapted version of the “EuroGuiDerm Guideline on lichen sclerosus” by Kirtschig G. et al.^1^, ^2^ The translated guideline was supplemented specifically by key contents and aspects particularly relevant for the healthcare condition in German‐speaking countries.

The present publication is a short version of the guideline. Several chapters are not included in this short version. In some parts, the background text of the included chapters is considerably shortened. The complete long version, guideline development report and evidence report are available on the AWMF website: https://register.awmf.org/de/leitlinien/detail/013‐105.

## METHODS

Further information is available in the guideline development report or the long version of the guideline.

The terminology and symbols used for standardized presentation of the recommendations are presented in Table 1. All recommendations were newly formulated, discussed and consented for the German AWMF S3 guideline.

**TABLE 1:** Recommendation strengths – wording, symbolism, and interpretation, modified according to Kaminski‐Hartenthaler et al.^3^

Recommendation strength	Wording	Symbol	Interpretation
*Strong* recommendation for the use of an intervention	“we recommend …”	**↑↑**	We believe that all or almost all informed people would make that choice. Clinicians will have to spend less time on the process of decision‐making, and may devote that time to overcome barriers to implementation and adherence. In most clinical situations, the recommendation may be adopted as a policy.
*Weak* recommendation for the use of an intervention	“we suggest…”	**↑**	We believe that most informed people would make that choice, but a substantial number would not. Clinicians and health care providers will need to devote more time on the process of shared decision‐making. Policy makers will have to involve many stakeholders and policy making requires substantial debate.
*Open* recommendation	“…may be considered…”	⇔	At the moment, a recommendation in favor or against an intervention cannot be made due to certain reasons (e.g., no reliable evidence data available, conflicting outcomes, etc.)
*Weak* recommendation against the use of an intervention	“we suggest against…”	**↓**	We believe that most informed people would make a choice against that intervention, but a substantial number would not.
*Strong* recommendation against the use of an intervention	“we recommend against…”	**↓↓**	We believe that all or almost all informed people would make a choice against that intervention. This recommendation can be adopted as a policy in most clinical situations.

## DEFINITION OF DISEASE

Lichen sclerosus (LS) is an inflammatory skin disease that typically involves the anogenital region where it causes itching and pain. The disease may lead to sexual and urinary dysfunction in women and men; however, it may be asymptomatic. Early symptoms include urological complaints, dyspareunia, and postcoital rhagades.^4^, ^5^ First signs of LS are usually a whitening of the genital skin, sometimes redness and edema; fissuring, scarring, atrophy, and fusion of structures may follow in its course (Table 2). In adulthood, LS is associated with an increased risk of local cancer, usually squamous cell carcinoma. Extragenital disease occurs in a minority of patients. The course of LS is usually chronic. Treatment remains often unsatisfactory, particularly if initiation of adequate treatment is delayed, given that impairing sclerosis of the skin may occur despite treatment.^6^, ^7^, ^8^, ^9^ There is some evidence that LS in males may go into remission after circumcision, although thorough studies are lacking.

**TABLE 2:** Symptoms and clinical signs of lichen sclerosus.

− **Symptoms** − Itch (mainly in genital LS in females) − Pain/burning − Irritation − Feeling of dryness − Dysesthesia − Constipation, in perianal involvement, particularly in girls − Dyspareunia or apareunia (disturbed sexual functioning) − Dysuria (pain, disturbed urinary stream) − Urinary bladder pain (abacterial cystitis) − LS can be asymptomatic	− **Clinical signs** − Edema (swelling of the skin) − Slight erythema (redness) − Hyperkeratosis (white thickened skin; hyperkeratosis on histology) − Sclerosis (tight, yellowish white skin, e.g. resulting in phimosis; dermal hyalinization on histology) − Pallor (pale, whitish areas; the histological correlate is not described) − Atrophic skin (crinkly skin; epidermal atrophy on histology) − Fissuring (skin fragility, loss of elasticity leading to splitting of skin) − Erosions − Blistering is very rare − Ecchymoses/purpura is common in genital LS (due to fragile, sclerotic and ectatic blood vessels) − Changes may be localized to the vulva or include the perianal area, forming a “figure‐of‐eight” distribution − Scarring may lead to architectural changes (e.g., resorption of the labia minora, fusing in the midline with burying, but not loss of the clitoris in women and e.g. phimosis, a narrow meatus urethrae and a sclerotic frenulum breve in men − Follicular plugging

Synonyms like kraurosis vulvae, balanitis xerotica obliterans, and white spot disease are outdated terms and should no longer be used. The suffix *et atrophicus* has been dropped because some cases of LS are associated with a hypertrophic, rather than atrophic, epithelium.

LS has a significant impact on the quality of life of affected patients. It is, therefore, important to raise more awareness of this not uncommon disease in order to diagnose and treat it early.^10^, ^11^, ^12^

## CLINICAL PRESENTATION AND SEQUEL OF DISEASE

LS is a chronic disease with diverse and varying symptoms. In girls and women, itch or pain is the main initial complaint in genital LS, while men rather complain about sexual dysfunction.^4^

### Clinical presentation in girls and women

**FIGURE 1:**
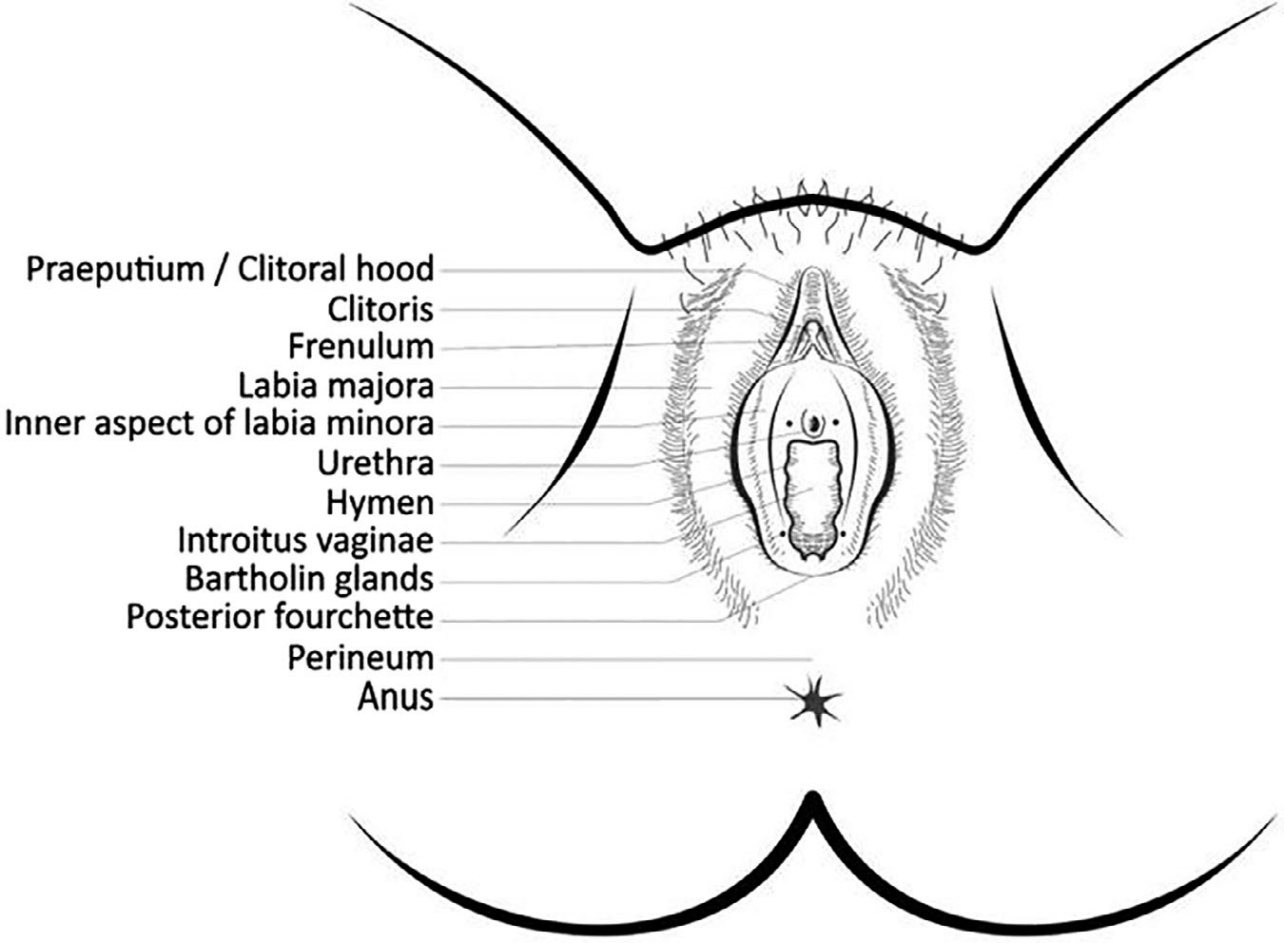
Graph of female external genitalia adopted from Gynecologic Dermatology.^13^

**FIGURE 2:**
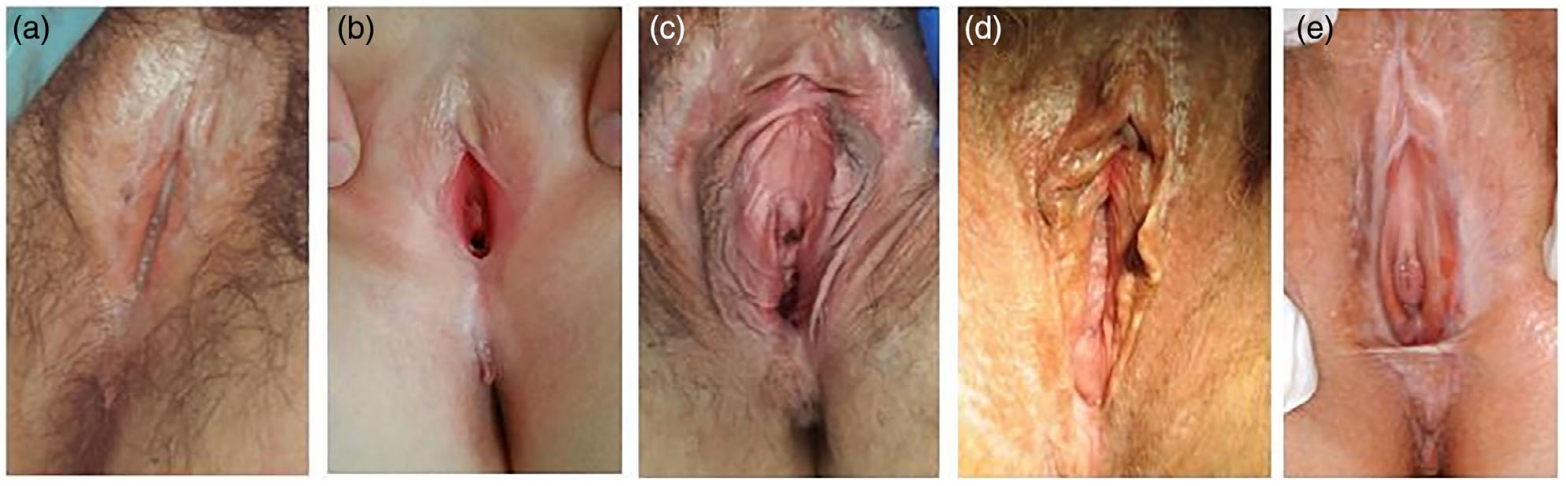
Typical changes of female external genitalia in lichen sclerosus: (a) Lichen sclerosus in two (pre‐)pubertal girls showing severe hyperkeratosis around the clitoris and interlabial sulci.^14^ (b) Mild hyperkeratosis around the clitoris and posterior fourchette. (c) Early lichen sclerosus without any sclerotic changes; patient complained of itch and fissuring during intercourse, shortened left labium minus. (d) White lesions around the clitoris and hyperkeratosis at the posterior fourchette. (e) Fusion of labia minora above the clitoris resulting in a buried clitoris and atrophic labia minora as manifestation of architectural changes; ecchymosis at the left interlabial sulcus.

The characteristic sites involved in females are the interlabial sulci, labia minora, glans clitoris, clitoral hood, posterior fourchette, and perineum (often in girls). Labia majora and the urethral meatus may also be affected (Figures 1, 2).

### Clinical presentation in boys and men

**FIGURE 3:**
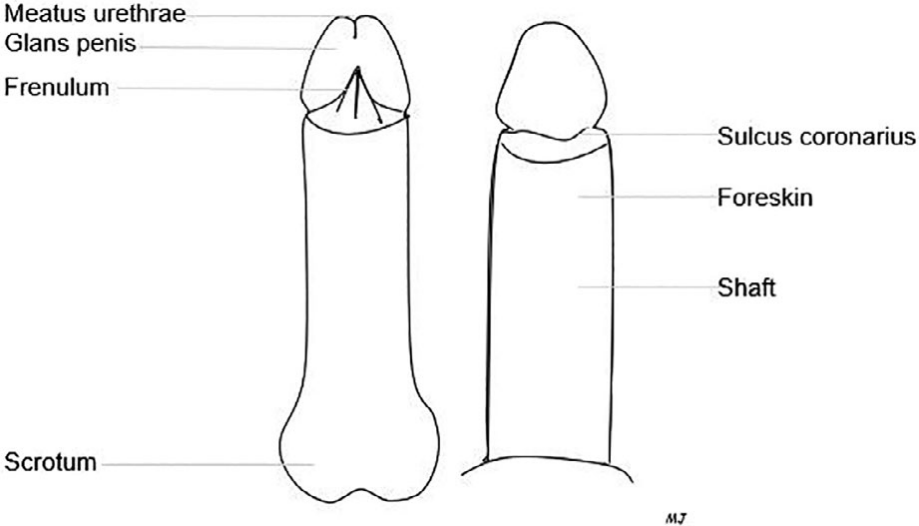
Graph of penile structures.

**FIGURE 4:**
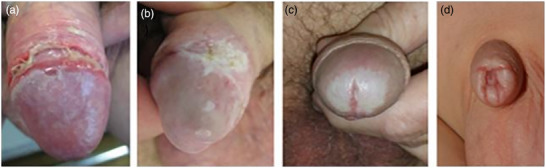
Lichen sclerosus in boys and men. (a) Erosive lichen sclerosus at the sulcus coronarius and white lesions at the glans penis. (b) Hyperkeratotic plaque at the sulcus. (c) Pallor or sclerosis at the glans with the meatus affected by lichen sclerosus. (d) Secondary phimosis in a boy.

LS in men and boys usually occurs on the glans penis, coronary sulcus, urethral meatus, and/or foreskin, with a predilection for the perifrenular area (Figures 3, 4). This may cause phimosis in a previously retractable foreskin or adhesions of the foreskin to the glans causing dysuria or painful erection. Secondary phimosis is highly suspicious to be caused by LS, especially in children. Rarely, penile shaft, perineal, scrotal, or perianal skin are affected. Meatal stenosis may lead to problems passing urine. Urethral involvement presents a severe complication of LS. LS in men is thought to more frequently affect uncircumcised or late circumcised men compared to those circumcised shortly after birth.^15^, ^16^

### Clinical presentation of extragenital LS

**FIGURE 5:**
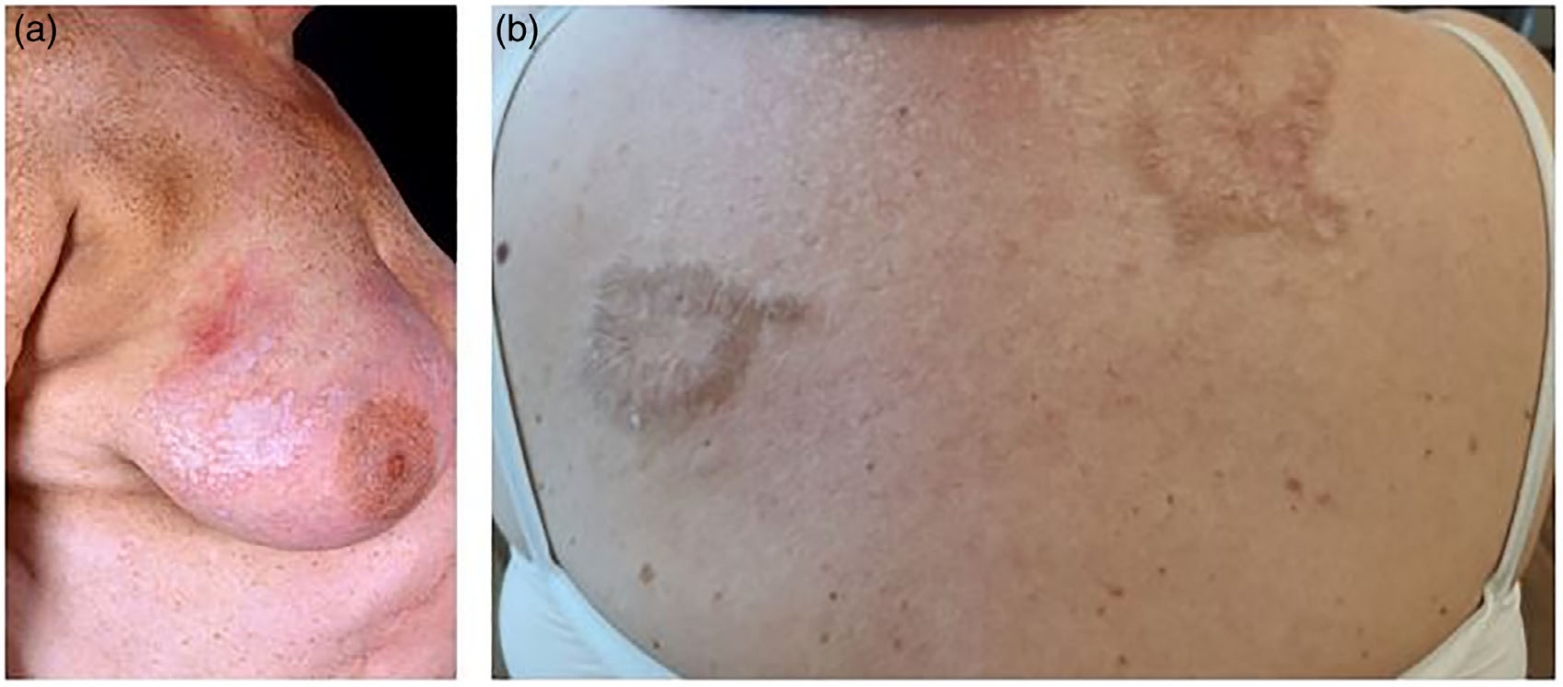
Extragenital lichen sclerosus. (a) Sclerotic lesion at the breast. (b) Sclerotic and hyperkeratotic lesion at the back.

LS of the extragenital skin alone is rare and has been reported in about 6% of all affected women (Figure 5).^17^ Involvement of the scalp, including bullous variants and scarring alopecia, is rare.^18^, ^19^, ^20^ Involvement of oral mucosa and nails has been reported in extremely rare cases.^21^, ^22^, ^23^, ^24^, ^25^, ^26^

In contrast to the usually genital manifestation of LS in the Western hemisphere, an Iraqi group reported approximately 10% genital manifestation and 90% extragenital lesions affecting predominantly extremities and trunk, but also lips and scalp.^27^

### Symptoms and clinical signs of LS

Physicians should be familiar with the various clinical signs of LS. Some of the clinical signs represent early, reversible signs while others are permanent, non‐reversible signs of LS.

*Reversible features*:

− Fissures/erosions are longitudinal ruptures/superficial wounds of the skin surface.
− Ecchymoses are bleedings within the skin.
− Hyperkeratoses are plaques of bright white skin with a “powdery” appearance.

*Irreversible features*:

− Sclerosis are areas of yellowish/ivory white skin with a smooth/waxy/firm texture.
− Pallor or pale whitish skin differs from hyperkeratosis in that the areas are not “powdery”.

*Complications*:

− loss of self‐esteem (for example, concern about the genital appearance),
− impaired quality of life,
− anxiety and mental health disorders,
− social loss (for example, partner),
− development of anogenital carcinoma (actual risk < 5%),
− development of clitoral pseudo‐cyst,
− sexual dysfunction,
− urinary dysfunction,
− genital dysesthesia.

## DIAGNOSIS

The diagnosis of LS is usually made based on the characteristic clinical appearance. While clinical scores are in development, they are not yet established in practice.^4^, ^28^, ^29^, ^30^, ^31^, ^32^, ^33^, ^34^

In typical cases, a biopsy is not needed, for example, if hyperkeratosis or sclerosis and ecchymoses are apparent. Taking a biopsy may, however, be useful at first presentation if the clinical diagnosis is not conclusive. In children, this is usually avoided, because it may be very traumatic for the child. However, a biopsy should be considered, also in children, if the clinical diagnosis is uncertain, dysplasia/malignant disease is suspected, or if there is failure of first‐line treatment. In the latter case, verification of therapy adherence is essential. In boys with secondary phimosis, circumcision is usually performed, which is followed by histological examination of the foreskin. Accordingly, a biopsy is irrelevant.

If a biopsy is taken, clinical‐pathological correlation is essential especially in early disease, given that histology can be non‐specific at this stage.

### Histopathology

If a biopsy is required for diagnosis, it should be taken from an untreated and typical lesion with whitish appearance (hyperkeratosis, “pallor”, or sclerosis). If these cannot be found, a biopsy may be taken, if fissures or erosions are apparent, at the end of a fissure, typically appearing in the interlabial sulcus, or at the edge of an erosion (not from the middle of an erosive lesions). If the labia minora are shortened, which may indicate disease activity, a biopsy can be taken at their caudal end.

If no biopsy was taken before initial treatment, a three‐week pause in treatment is recommended (skin care products are allowed) for a reliable histological diagnosis. If this pause in treatment is not tolerated by the patient, it is essential to inform the pathologist about the type of pretreatment, given that histological features may change depending on the length and type of treatment.

However, it has to be noted that there is a spectrum of histological and clinical features in LS. This may cause false negative histological results. In the early phase of the disease, some features present in persistent disease may be absent, for example, the hyalinization of the upper dermis may be lacking, thus impairing (also) a reliable histological diagnosis. In some cases, hypertrophic forms of genital LS and lichen planus (LP) may show similar clinical and histological features making their differentiation difficult. It is important to rule out precancerous or cancerous lesions. Squamous cell carcinomas (SCCs) in LS develop independent of human papilloma virus (HPV) infection. They can develop within several months.^35^ Therefore, any new hyperkeratotic lesion or newly arising erosions and ulcerations are suspicious for an HPV‐independent vulval (and penile) intraepithelial neoplasia, also referred to as differentiated vulval intraepithelial neoplasia. These should always be biopsied.

In conclusion, greater awareness of the clinical and histological spectrum of LS should enable earlier diagnosis and treatment. A biopsy read by an experienced (dermato)histopathologist is helpful to rule out clinical differential diagnoses, such as LP and eczema (atopic or seborrheic), in particular if early LS is suspected, and to detect precancerous and cancerous lesions.

### Diagnosis in children

Special attention has to be paid to children with anogenital disease and it has to be kept in mind that children are not *little adults*.

A child‐friendly setting should be created when examining and treating children. The investigation should be performed by experienced physicians who are familiar with the disease, the anatomy of the genitals in children, and who know how to communicate with children.

Preferentially, the investigation of girls should be performed using a patient couch and not a gynecological chair to provide a child‐oriented examination environment.

## INTRODUCTION INTO THERAPEUTIC MANAGEMENT

### Aims of treatment

Apart from individual treatment goals, the therapeutic approach should in general be multidimensional and aim at:

1. Rapid improvement of symptoms, such as pruritus, pain, or burning sensation.
2. Maintenance or improvement of quality of life including sexual life and voiding.
3. Control of clinical signs of disease to avoid, for example, scarring, resorption of tissue, dermal atrophy, and malignant transformation.
4. Reduction of flare‐ups.
5. Cure of LS in boys and men.

For each patient, the aims of treatment have to be assessed individually. In the course of the disease, these aims have to be reassessed from time to time.

## SKIN CARE AND GENERAL TREATMENT RECOMMENDATIONS

**Table:** 

We *recommend* the use of topical ointments instead of creams or gels in LS patients.	**↑↑**	100% consensus consensus‐based

**Table:** 

We *suggest* avoidance of trigger factors (mechanical irritation, such as trauma, unnecessary surgical interventions, piercings) and irritants (for example, excessive water exposure or excessive use of cleansing products, synthetic and tight clothing, use of wet wipes) at the sites affected by LS.	**↑**	100% consensus consensus‐based

**Table:** 

We *suggest* regular change of incontinence pads and undergarments and the best possible management of urine incontinence in LS patients.	**↑**	100% consensus consensus‐based

**Table:** 

We *suggest against* the application of herbal topical products, topical antihistamines and anesthetics, and perfumed topical products due to an increased risk of inducing contact sensitization in LS patients.	**↓**	> 75% consensus consensus‐based

**Table:** 

We *recommend* assuring adherence to adequate treatment and considering ruling out other causes, such as contact allergies, infections, and malignancy if the clinical picture of LS worsens or symptoms such as itch or pain increase.	**↑↑**	100% consensus consensus‐based

## TOPICAL THERAPY WITH EMOLLIENTS

**Table:** 

We *recommend* basic treatment with emollients in addition to standard therapy in *women and girls* with genital LS.	**↑↑**	100% consensus consensus‐based*
We *suggest* basic treatment with emollients in addition to standard therapy in *men and boys* with genital LS.	**↑**	
We *suggest* basic treatment with emollients in addition to standard therapy in patients with *extragenital LS*.	**↑**	
*Based on the low level of evidence, the guideline development group decided to provide purely consensus‐based recommendations despite systematic review of evidence (see evidence report).		

### Efficacy and mechanism of action

Emollients are pharmaceutical products making the skin soft and supple. They act by “filling” the gaps between corneocytes. This “sealing” of the skin results in a lower transdermal loss of moisture. Subjectively, people experience less dryness and a smoother, softer skin by applying emollients. This effect has been demonstrated, in particular, in people with certain dermatoses, such as atopic dermatitis and psoriasis vulgaris.

Examples of emollients include: lanolin, fatty acids and their derivatives, specifically, paraffins processed to petroleum jelly, vegetable oils, but also propylene glycol derivatives.

Emollients may give additional symptom relief in LS after initial treatment with topical corticosteroids.

A trial with initial application of a topical corticosteroid followed by maintenance treatment with daily application of *cold cream* in women with LS showed that initial symptom relief was maintained during maintenance therapy. However, it is not possible to extrapolate that this was the effect of emollients or a long‐term effect of the corticosteroid.^36^

Given that a randomized trial of topical vitamin E cream compared to emollient following an initial treatment with topical corticosteroid showed similar recurrence rates over a one‐year period, vitamin E does not appear to have any advantage over emollients.^37^

### Dosage: acute and maintenance

Currently, no data are available regarding the optimal dosage. Based on our experience, we recommend applying emollients at least two times a day to improve the skin barrier and protect it from irritating external factors, such as urine, soaps, vaginal discharge, sweat, lubricants, semen, and friction during sexual intercourse. In case of vulval burning during urination, application of emollients prior to urination might help to reduce skin contact with urine. However, to avoid a diluting effect of topical corticosteroids, emollients and corticosteroids should not be applied simultaneously.

### Safety and special considerations

Generally, emollients are very well tolerated, and no safety concerns have been raised. However, in rare cases they may lead to irritant or allergic contact dermatitis. For example, additives, such as benzoic acid, benzalkonium chloride, polyethylene glycol or sodium lauryl sulphate (SLS), are known irritants^38^, ^39^ that may be ingredients of emollients. Likewise, benzoic acid may act as an allergen in emollients. This applies also to lanolin, jojoba oil, propylene glycol fragrances, and balsam of Peru belonging to the most frequent allergens.^38^, ^40^ Therefore, only fragrance‐free emollients should be used.

## TOPICAL AND INTRALESIONAL THERAPY WITH CORTICOSTEROIDS

**Table:** 

We *recommend* topical treatment with potent or ultrapotent corticosteroids in *women* with genital LS.	**↑↑**	100% consensus evidence‐ and consensus‐based, see evidence report
We *recommend* topical treatment with potent* or ultrapotent* corticosteroids in *girls* with genital LS.	**↑↑**	
We *recommend* topical treatment with potent or ultrapotent corticosteroids in *men* with genital LS.	**↑↑**	
We *recommend* topical treatment with potent* or ultrapotent* corticosteroids in *boys* with genital LS.	**↑↑**	
We *suggest* topical treatment with potent* or ultrapotent* corticosteroids in patients with *extragenital LS*.	**↑**	
*Not all substances are approved for children of every age group.		

**Table:** 

We *recommend* the use of topical corticosteroid ointments instead of creams or lotions in patients with LS.	**↑↑**	100% consensus consensus‐based

**Table:** 

In *women* with hyperkeratotic genital LS, treatment with intralesional corticosteroids *may be considered* for LS lesions resistant to topical therapy (provided neoplasia has been excluded).	⇔	100% consensus evidence‐ and consensus‐based, see evidence report
We *recommend against* treatment with intralesional corticosteroids in *girls* with genital LS.	**↓↓**	
In *men* with hyperkeratotic genital LS, treatment with intralesional corticosteroids *may be considered* for LS lesions resistant to topical therapy (provided neoplasia has been excluded).	⇔	
In *boys* with genital LS, treatment with intralesional corticosteroids *may be considered* in the context of foreskin‐preserving surgery.	⇔	

### Potent and ultrapotent topical glucocorticoids

#### Efficacy and mechanism of action

Based on their proven efficacy and safety, potent (class III) or ultrapotent (class IV) topical corticosteroids like mometasone furoate 0.1% or clobetasol propionate 0.05% ointment (or cream) are recommended as preferred treatment, both in acute episodes and in maintenance therapy.^41^, ^42^, ^43^

Topical corticosteroids function as anti‐inflammatory and anti‐fibrotic agents. They influence multiple different signaling pathways by inhibiting expression of activated (pro)‐inflammatory genes.^44^, ^45^, ^46^ In addition, they have a rapid anti‐pruritic effect.

Potent or ultrapotent topical corticosteroids rapidly improve clinical signs and subjective symptoms (usually in less than 10 days).

Creams and ointments are the most frequently used vehicles as they spread easily and adhere adequately to mucosal surfaces. According to our experience, however, several different types of vehicles should be tested by the patient to find the most efficacious and comfortable treatment formulation as this will help to maintain adherence. It should be noted, however, that ointments are usually less stinging, contain less contact allergens, provide a better barrier protection and usually allow for a better penetration of the active ingredient into the skin than creams.

The efficacy of potent or ultrapotent topical corticosteroids appears to be comparable in the treatment of genital LS. Most studies have been performed with ultrapotent corticosteroids (clobetasol propionate 0.05%). However, modern potent corticosteroids such as mometasone furoate may be preferred in certain situations. This applies to children because the skin is thinner and steroid‐related side effects may occur more frequently with ultrapotent steroids. Moreover, potent corticosteroids may be preferred in pregnancy where resorption of the steroid should be avoided because of safety aspects (risk of growth retardation).

#### Girls and women with genital LS

About 60% to 70% of female LS patients achieve complete remission of their symptoms after a three‐month course of clobetasol propionate 0.05%, usually applied once daily.^8^, ^47^, ^48^ Similarly effective was mometasone furoate 0.1% once daily after 12 weeks in a head‐to‐head trial. 59% and 37% of patients in the clobetasol propionate group and 67% and 48% in the mometasone furoate group achieved an improvement of at least 75% in subjective and objective scores, respectively.^47^, ^48^

There is evidence to suggest that less potent corticosteroids (for example, triamcinolone and prednicarbate) are also effective as maintenance therapy or for the treatment of moderate episodes or recurrences.^45^, ^49^, ^50^

Although there are no comparative randomized trials in girls with LS, non‐comparative studies show that treatment with potent or ultrapotent topical corticosteroids is effective in suppressing clinical signs and symptoms of LS.^51^, ^52^ Although LS will go into remission after childhood in some patients, the course is variable and close follow‐up during and after puberty is required to detect recurrence of LS early.^53^

#### Boys and men with genital LS

Both mometasone furoate 0.1% and clobetasol propionate 0.05% are effective in treating early and intermediate penile LS, but the rate of cure is unknown. A placebo‐controlled randomized trial assessed the efficacy of topical mometasone furoate 0.05% ointment in 40 boys with penile LS after five weeks of application. Mometasone furoate 0.1% improved the clinical grade of phimosis in 7 out of 17 boys. No improvement was observed in advanced cases.^54^

Patients with penile LS are at risk of developing penile cancer.^55^, ^56^, ^57^

#### Dosage: acute and maintenance treatment

In clinical trials, clobetasol propionate 0.05% cream or ointment was applied once or twice daily for 3 months, or tapered gradually. For example, it was applied once or twice daily for one month then once daily or on alternate days for 2 months. In some cases, it was applied depending on the severity of clinical signs and symptoms and the age of the patient.^41^, ^48^, ^58^

There is no consensus on a general standard dosage regimen for the treatment of LS (compare regimens mentioned above).

Only in rare instances is there a complete remission without recurrence.

There is evidence that less potent corticosteroids (for example, triamcinolone and prednicarbate) are effective as maintenance therapy and for the treatment of moderate episodes or recurrences.^45^, ^49^, ^50^

#### Safety

Not all substances are approved for children of every age group. A potential off‐label use of topical corticosteroids should be discussed with the patients and their parents.

Adverse effects of topical corticosteroids are uncommon. Rarely, local irritation and burning, especially during the first treatment applications and if the skin is particularly inflamed, are experienced. This is more often observed when creams instead of ointments are used. In the long term, dryness, hypopigmentation, and dermal atrophy may be observed, particularly in keratinized skin. However, topical corticosteroids can be applied in LS over years without significant clinically relevant adverse effects (often, 30 g to 100 g clobetasol propionate 0.05% are sufficient for the entire year during maintenance phase; in acute phases, 30 g per month should not be exceeded). The adverse effects like stinging, burning, and xerosis are most commonly linked to the vehicle and not to the steroid itself.^59^

However, it is important to point out where the topicals have to be applied and how much has to be used. For example, one fingertip unit is sufficient to treat the whole vulva. It has not been studied whether topical corticosteroids have to be applied only to areas that are visibly affected by LS, for example, only to clitoris, labia minora, interlabial sulci, or perineum, or to the entire anogenital area that may be affected by LS. The hair‐bearing labia majora, for example, should certainly not be treated, given that they are virtually never affected by LS. Topical corticosteroids must not be applied to unaffected skin where they will cause adverse effects like erythema, irritation and dermal atrophy if continuously used.

### Intralesional glucocorticoids

Intralesional injection of triamcinolone acetonide or dexamethasone may be an alternative treatment to ultrapotent topical corticosteroids for some patients with LS.^60^, ^61^, ^62^

Intralesional corticosteroid injections may be tried if there is a lack of response to potent or ultrapotent topical corticosteroids if, for example, poor penetration (for example, in very hyperkeratotic lesions) or a lack of compliance is suspected.^61^ Intralesional corticosteroid injections should be avoided in atrophic skin or in small lesions as the tissue may become damaged and ulcerate. The therapy should be conducted and monitored by a physician with experience in intralesional injection of corticosteroids.

## TOPICAL THERAPY WITH CALCINEURIN INHIBITORS

**Table:** 

We *suggest* topical calcineurin inhibitors (off label) in *women* with genital LS as second choice or as additional topical treatment if topical corticosteroids are contraindicated or insufficient.	**↑**	100% consensus evidence‐ and consensus‐based, see evidence report
We *suggest* topical calcineurin inhibitors (off label) in *girls* with genital LS as second choice or as additional topical treatment if topical corticosteroids are contraindicated or insufficient.	**↑**	
We *suggest* topical calcineurin inhibitors (off label) in *men* with genital LS as second choice or as additional topical treatment if topical corticosteroids are contraindicated or insufficient.	**↑**	
We *suggest* topical calcineurin inhibitors (off label) in *boys* with genital LS as second choice or as additional topical treatment if topical corticosteroids are contraindicated or insufficient.	**↑**	
In patients with *extragenital LS*, treatment with topical calcineurin inhibitors (off label) *may be considered*.	⇔	

### General introduction

Two topical calcineurin inhibitors (TCIs), pimecrolimus 1% cream and tacrolimus 0.1% and 0.03% ointment, are used *off label* to treat LS.^63^, ^64^, ^65^, ^66^, ^67^, ^68^, ^69^, ^70^, ^71^, ^72^, ^73^, ^74^, ^75^, ^76^, ^77^ Currently, these drugs are only approved for the treatment of atopic dermatitis. There are few randomized studies comparing TCIs with clobetasol propionate 0.05% in vulval LS.^69^, ^78^, ^79^

### Efficacy and mechanism of action

Tacrolimus is a lipophilic immunosuppressant that inhibits the *second messenger* calcineurin and blocks the transcription of proinflammatory cytokines, such as interleukin (IL)‐2 and interferon‐gamma. By inhibiting calcineurin, tacrolimus also reduces antigen presentation and T‐cell activation. Moreover, it affects other cell types involved in pruritus and inflammation, such as mast cells, eosinophils, and basophils by inhibition of IL‐3, IL‐8, IL‐13, and granulocyte‐macrophage colony‐stimulating factor (GM‐CSF). On epidermal antigen‐presenting cells, FcεR1 receptor expression is reduced. While many of the pharmacological effects of topical calcineurin inhibitors are similar to those of corticosteroids, side effects, such as dermal atrophy and telangiectasia, are not observed with topical tacrolimus and pimecrolimus.^67^, ^68^

### Women with genital LS

In a double‐blind, randomized trial of 38 women with histologically confirmed vulval LS, the patients were treated for 12 weeks with either topical pimecrolimus 1% or clobetasol propionate 0.05%. Both groups showed similar improvement in pruritus and pain.^78^ Clobetasol propionate 0.05% was found to be superior in reducing inflammation (p = 0.015).

The relief of LS symptoms by pimecrolimus was also confirmed by several case series.^64^, ^80^, ^81^

A multicenter phase II trial assessed the safety and efficacy of tacrolimus ointment 0.1% for the treatment of LS.^68^ Eighty‐four patients (49 women, 32 men and 3 girls) between 5 and 85 years with long‐standing, active LS (79 with anogenital and 5 with extragenital LS) were treated twice daily for 16 weeks. Fourteen patients dropped out early. Remission of active LS was reached in 43% (ITT 36%) of patients after 24 weeks of treatment. Partial remission was observed in 34% (ITT 29%) of patients. Maximal effects occurred between weeks 10 and 24 of therapy.

### Men with genital LS

In a retrospective analysis, Kyriakou et al. concluded that clobetasol propionate 0.05% cream is effective in the treatment of genital LS in males. During the subsequent maintenance therapy with methylprednisolone aceponate 0.1% cream or tacrolimus 0.1% ointment, no difference between the two substances was observed concerning the recurrence rate.^82^

### Children with genital LS

There are few case series reporting treatment of LS with topical tacrolimus in children. In smaller studies and case reports, application of 0.03%^83^, ^84^ or 0.1%^85^ tacrolimus ointment achieved clinical improvement up to complete remission in prepubertal girls and boys. The recurrence rate was reduced by maintenance therapy with decreased application frequency for several months.^84^ Short‐term adjuvant tacrolimus treatment was also effective and well tolerated after surgical interventions in boys with histologically confirmed LS.^86^

### Extragenital LS

The sole use of topical tacrolimus for extragenital LS proved unsuccessful^73^, ^74^ or inferior to topical corticosteroids.^69^ However, extragenital LS has been treated successfully with tacrolimus combined with UV light.^74^, ^82^, ^87^

### Dosage: acute and maintenance

There is no consensus on the treatment regimen of genital LS with topical calcineurin inhibitors. The usual approach is to apply topical calcineurin inhibitors initially twice daily possibly followed by once daily as soon as the lesions regress, for 3 to 6 months continuously.^63^, ^65^

In a retrospective analysis, Anderson K et al. reported that topical calcineurin inhibitors may be used for maintenance therapy in girls after initial treatment with topical clobetasol propionate 0.05% or mometasone furoate 0.1% once or twice daily for several weeks. In general, *off‐label* de‐escalation of topical corticosteroids to topical tacrolimus can be recommended in patients with LS. Topical corticosteroids may be tapered to once daily application once a week while topical tacrolimus 0.1% or, in children, 0.03% is used once daily. With maintained clearance of lesions, clobetasol propionate 0.05% application may be discontinued and tacrolimus tapered to once daily 1–2 times a week.^88^

### Safety

Topical calcineurin inhibitors do not induce dermal atrophy, hypopigmentation, striae, telangiectasias, rebound flares, or hypothalamic‐pituitary‐adrenal axis suppression. The large molecular size of topical calcineurin inhibitors minimizes their absorption through the skin into the circulation. Therefore, their long‐term use is associated with minimal systemic absorption with no evidence of systemic accumulation in pharmacokinetic studies on adult and pediatric patients with atopic eczema.^75^ The blood concentrations of pimecrolimus were checked in ten patients and were undetectable in all cases.^64^

Infections such as genital herpes and vulvovaginal candidiasis occurred in 2% of 84 patients treated with tacrolimus. No malignancy was observed during an 18‐month follow‐up period.^68^ The bacterial vaginosis observed in a case report of a 10‐year‐old girl with LS has been associated with the immunosuppressive effect of topical tacrolimus 0.1%.^89^

The theoretical safety concern (observed in an animal study) that topical calcineurin inhibitors may increase the risk of lymphoma and other cutaneous malignancies is not supported by case‐control‐studies, meta‐analyses, and post marketing registries.^76^, ^77^

### TOPICAL THERAPY WITH RETINOIDS

**Table:** 

In *women* with genital LS, treatment with topical retinoids (off label) *may be considered*.	⇔	100% consensus evidence‐ and consensus‐based, see evidence report
In *girls* with genital LS, treatment with topical retinoids (off label) *may be considered*.	⇔	
In *men* with genital LS, treatment with topical retinoids (off label) *may be considered*.	⇔	
In *boys* with genital LS, treatment with topical retinoids (off label) *may be considered*.	⇔	
In patients with *extragenital LS*, treatment with topical retinoids (off label) *may be considered*.	⇔	

### Introduction

Retinoids induce changes in both dermis and epidermis. Many of their effects are mediated by their interaction with nuclear retinoic acid receptors (RARs) and retinoid X receptors (RXRs).^90^ An imbalance in the expression of nuclear RARs (RAR‐α and RAR‐γ) has been postulated to be involved in the pathogenesis of vulval LS.^91^

### Efficacy and mechanism of action

There are only few case series reporting topical retinoids in the treatment of LS.

The evidence concerning the efficacy of topical retinoids in treatment of vulval LS is limited and based primarily on small case series and uncontrolled studies. In an open study on 22 patients, one‐year application of 0.025% tretinoin resulted in an improvement of subjective symptoms, such as pruritus, burning sensations, and dyspareunia, as well as objective findings (for example, hyperkeratosis, sclerosis).^92^ In another case series, 13‐cis retinoic acid (0.5%) and 0.025% tretinoin resulted in complete or partial remission of symptoms.^93^, ^94^ A retrospective cohort study revealed no additional benefit of the combination of tretinoin with mometasone furoate compared to mometasone furoate alone.^95^

### Safety

Side effects, mainly mild erythema and burning, are reported in about 35% of patients.^94^ Rarely, patients discontinued treatment due to adverse effects.

### Special aspects

Topical retinoids are contraindicated during pregnancy or if pregnancy is planned. If topical retinoids are used by women of child‐bearing age, they must be discontinued if pregnancy occurs, and ultrasound examination checking specifically for malformations should be offered.

### TOPICAL THERAPY WITH HORMONES

**Table:** 

We *recommend against* topical *testosterone* or *dihydrotestosterone* in *women* as a treatment for genital LS.	**↓↓**	100% consensus evidence‐ and consensus‐based, see evidence report
We *recommend against* topical *progesterone* in *women* as a treatment for genital LS.	**↓↓**	
We *recommend against* topical *estrogen* in *women* as a treatment for genital LS.*	**↓↓**	
We *recommend against* topical hormone preparations in *girls* as a treatment for genital LS.	**↓↓**	
We *recommend against* topical hormone preparations in *men* as a treatment for genital LS.	**↓↓**	
We *recommend against* topical hormone preparations in *boys* as a treatment for genital LS.	**↓↓**	
We *recommend against* topical hormone preparations in patients as a treatment for *extragenital LS*.	**↓↓**	
*Topical vaginal estrogen treatment may, however, be useful in women with additional genitourinary syndrome.		

### PLATELET‐RICH PLASMA

**Table:** 

In *women* with genital LS, treatment with platelet‐rich plasma *may be considered*.	⇔	> 75% consensus evidence‐ and consensus‐based, see evidence report
We *recommend against* platelet‐rich plasma in *girls* as a treatment for genital LS.	**↓↓**	
In *men* with genital LS, treatment with platelet‐rich plasma *may be considered*.	⇔	
We *recommend against* platelet‐rich plasma in *boys* as a treatment for genital LS.	**↓↓**	
We *recommend against* platelet‐rich plasma in patients as a treatment for *extragenital LS*.	**↓↓**	

### UV THERAPY

**Table:** 

UV therapy *may be considered* in *women* with genital LS.	⇔	100% consensus evidence‐ and consensus‐based, see evidence report
We *recommend against* UV therapy in *girls* as a treatment for genital LS.	**↓↓**	
UV therapy *may be considered* in *men* with genital LS.	⇔	
We *recommend against* UV therapy in *boys* as a treatment for genital LS.	**↓↓**	
We *recommend* UV therapy in patients with *extragenital LS*.	**↑↑**	

### Efficacy and mechanism of action

Phototherapy, especially with ultraviolet (UV) A, is an effective and well‐established treatment option for sclerosing skin diseases, such as morphea, skin involvement in systemic sclerosis, or sclerodermatous graft‐versus‐host disease. Numerous studies (including prospective controlled trials) have been performed for various sclerosing skin diseases.^96^, ^97^, ^98^, ^99^, ^100^ In contrast, only few data exist on the efficacy of UV therapy in genital LS. Most data are based on small case series or single case reports.^101^, ^102^, ^103^

### Dosage

UVA1 phototherapy should be used with low (20 J/cm^2^) or medium (50 J/cm^2^) dose for up to 40 applications per cycle.

### Safety

In general, UVA1 is well‐tolerated, side effects may include early erythema directly after irradiation, tanning of the irradiated skin, and pruritus or burning shortly after treatment.

### Monitoring

If possible, clinical examination during UVA1 treatment should be performed weekly or every 14 days.

### UV therapy in extragenital LS

Only few studies exist on the safety and efficacy of UV light in extragenital LS, too.^104^, ^105^, ^106^ The efficacy of UVA1 phototherapy in extragenital LS was first reported by Kreuter et al. in 2001.^105^ The authors reported improvement of LS‐related lesions in two patients after 40 sessions of UVA1 irradiation (four sessions per week for ten weeks, total of 40 treatments, 20 J/cm^2^ low‐dose UVA1 per session, 800 J/cm^2^ cumulative dose). One year later, Kreuter et al. were able to present the efficacy of UVA1 in extragenital LS in ten patients, all being treated by the established standard irradiation protocol.^106^, ^107^

As far as PUVA bath therapy in extragenital LS is concerned, one case report with a cumulative dose of 31.7 J/cm^2^ and single doses from 0.3 to 2.3 J/cm^2^ demonstrated a promising outcome.^108^

Narrowband (NB)‐UVB phototherapy alone or in combination with salt water (balneophototherapy) is a well‐established therapy for psoriasis. Similar to UVA1, only few case reports exist on NB‐UVB for extragenital LS.^104^, ^109^ However, a large randomized controlled trial in patients with morphea has shown that NB‐UVB has a positive effect on sclerotic skin lesions, although UVA1 was significantly more effective.^110^ Based on these findings, NB‐UVB therapy might be considered as an alternative treatment for extragenital LS in centers where UVA1 is not available. To our knowledge, the effective use of NB‐UVB in vulval LS has so far only been described in single case reports.^111^

Care has to be taken in patients with additional connective tissue diseases, such as lupus erythematosus, which may pose a contraindication for UV treatment.^112^

### PHOTODYNAMIC THERAPY

**Table:** 

Photodynamic therapy *may be considered* in *women* with genital LS.	⇔	100% consensus evidence‐ and consensus‐based, see evidence report
We *recommend against* photodynamic therapy in *girls* as a treatment for genital LS.	**↓↓**	
Photodynamic therapy *may be considered* in *men* with genital LS.	⇔	
We *recommend against* photodynamic therapy in *boys* as a treatment for genital LS.	**↓↓**	
Photodynamic therapy *may be considered* in patients with *extragenital LS*.	⇔	

### LASER THERAPY

**Table:** 

In *women* with genital LS, treatment with *fractionated ablative CO_2_ laser may be considered*.	⇔	100% consensus evidence‐ and consensus‐based, see evidence report
In *women* with genital LS, treatment with *non‐ablative Nd:YAG laser may be considered* in order to soften the tissue.	⇔	
In *men* with genital LS, treatment with *fractionated ablative CO_2_ laser may be considered*.	⇔	
In *men* with genital LS, treatment with *non‐ablative Nd:YAG laser may be considered* in order to soften the tissue.	⇔	
In patients with *genital LS*, *combination laser treatment* (for example, ablative and non‐ablative lasers) *may be considered*.	⇔	
We *recommend against* using laser treatment in *children* with LS.	**↓↓**	

### CRYOTHERAPY

**Table:** 

We *suggest against* cryotherapy in *women* as a treatment for genital LS.	**↓**	100% consensus evidence‐ and consensus‐based, see evidence report
We *recommend against* cryotherapy in *girls* as a treatment for genital LS.	**↓↓**	
We *recommend against* cryotherapy in *men* as a treatment for genital LS.	**↓↓**	
We *recommend against* cryotherapy in *boys* as a treatment for genital LS.	**↓↓**	
We *recommend against* cryotherapy in patients as a treatment for *extragenital LS*.	**↓↓**	

### SYSTEMIC THERAPY

**Table:** 

We *suggest acitretin* (off label), taking into account teratogenicity, if systemic therapy is needed in *women* with genital LS.	**↑**	100% consensus evidence‐ and consensus‐based, see evidence report
We *suggest* acitretin (off label) if systemic therapy is needed in *men* with genital LS.	**↑**	> 75% consensus evidence‐ and consensus‐based, see evidence report
We *suggest* methotrexate (off label), taking into account teratogenicity, if systemic therapy is needed in *adult patients* with *genital and/or extragenital LS*.	**↑**	100% consensus evidence‐ and consensus‐based, see evidence report

### Introduction

Systemic treatment of LS has been tried already with various substances. Overall, the level of evidence is very low and usually the drugs have not been tried in all forms of LS.

#### Systemic retinoids

#### Efficacy and mechanism of action

There are several case series as well as two randomized controlled trials reporting on the treatment of LS with oral retinoids. In an uncontrolled study, Mørk et al. observed an improvement of clinical symptoms (patients’ and physicians’ outcome assessment) in six of eight patients with treatment‐resistant vulval LS on oral etretinate (1 mg/kg/day) after 14–18 weeks.^113^ Romppanen et al. treated 19 women with vulval LS with oral etretinate for 3 months (initial dose 0.54 mg/kg/day, maintenance dose 0.26 mg/kg/day). A decrease in severity was achieved in nearly all cases among the group with severe vulval dystrophy.^114^ Furthermore, two small double‐blind, placebo‐controlled studies on the treatment of genital LS with acitretin have been published.^115^, ^116^ In a multicenter, double‐blind study, Bousema et al. treated patients (78 enrolled, 46 included in efficacy analysis) with vulval LS with 20 to 30 mg acitretin or placebo for a total of 16 weeks. Symptoms and clinical signs improved in both treatment group and placebo group. However, intensity of all symptoms and clinical signs was lower in the acitretin group, with statistically significant differences for pruritus, atrophy, and hyperkeratosis.^115^ In another randomized clinical trial by Ioannides et al., 52 male patients with severe, long‐standing LS were randomized in a 2:1 ratio to receive acitretin (35 mg) or placebo for 20 weeks. Mean total clinical score of the acitretin group was significantly lower than that of the controls, which was also accompanied by a significant improvement in mean DLQI.^116^ Based on these results the authors concluded that acitretin is effective in men with long‐standing LS.^115^

#### Safety

Retinoids are usually well tolerated, but dryness of skin and mucous membranes often occurs as a side effect. Cholesterol, triglycerides and transaminase levels can increase during therapy, and should be monitored regularly before and during therapy. Given that systemic retinoids are highly teratogenic, all women of childbearing age must use safe contraception (during therapy and, depending on the drug, for up to three years after cessation of therapy).

### Methotrexate

#### Efficacy and mechanism of action

Methotrexate (MTX) is a structural analog of folic acid and acts by inhibiting the metabolism of folic acid. It is used for the treatment of neoplasms and inflammatory diseases. A retrospective case series described 28 patients with LS (24/28 with extragenital involvement) who were treated with MTX 2.5 to 17.5 mg weekly. There was initial improvement of LS in 21/28 cases and sustained improvement in 15 cases. Most patients were treated in combination with topical corticosteroids or tacrolimus.^117^ In another trial, seven patients with generalized LS (five patients with genital plus extragenital involvement; two without genital involvement) were treated with high‐dose intravenous methylprednisolone, given as a 1000 mg single dose for three consecutive days monthly, plus MTX 15 mg/week (oral) for at least 6 months (maximum 10 months). All patients were previously treated unsuccessfully with topical corticosteroids and UV phototherapy. In all patients, extragenital LS improved after usually three months of treatment; 100% cure was not achieved. The effect on genital lesions was not reported. Adverse effects observed (nausea in three patients, headache in three patients, and a twofold increase of liver enzyme levels in one patient) were moderate and disappeared after the end of treatment.^118^ A patient with generalized LS involving the extragenital skin and anogenital site was successfully treated with MTX 10 mg/week for 8 months; improvement was noticed by 3 weeks and excellent response after 5 months.^119^

#### Dosage

Methotrexate between 10 and 15 mg/week (subcutaneous or oral) for 6 months, possibly combined with systemic corticosteroids, may improve treatment‐resistant generalized forms of LS.

#### Safety

The most common side effects are gastrointestinal problems, headache, fatigue, mood changes^120^, and elevated liver enzymes. Pancytopenia occurs mainly in case of overdose. MTX‐induced idiopathic pulmonary fibrosis has rarely been reported. Before starting MTX therapy, chronic infections (for example, hepatitis B/C, HIV, tuberculosis) must be excluded. Vaccinations following local guidelines are recommended. Live attenuated vaccines are contraindicated. Complete blood count as well as kidney and liver profile are mandatory before and regularly during therapy. National guidelines should be consulted.

## SURGICAL INTERVENTIONS

**Table:** 

We *suggest de‐adhesion / synechiolysis / perineoplasty, or anatomic vulvoplasty* in *women* with genital LS who have a persistent introital stenosis causing mechanical problems in voiding or sexual intercourse despite guideline‐conform treatment with topical corticosteroids.	**↑**	100% consensus consensus‐based*
We *suggest against* surgical interventions for treatment of genital LS in *girls*, although these may be indicated in individual cases to improve functional impairments.	**↓**	100% consensus consensus‐based*
We *recommend circumcision*, preferably removing the complete foreskin, in *men and boys* with phimosis due to LS that did not respond to guideline‐conform treatment with, for example, topical corticosteroids.	**↑↑**	> 75% consensus consensus‐based*
We *suggest frenuloplasty* in combination with local triamcinolone injection or follow‐up treatment with local corticosteroids in *men and boys* with isolated shortening of the frenulum due to LS that did not respond to guideline‐conform treatment with, for example, topical corticosteroids.	**↑**	100% consensus consensus‐based*
We *recommend urethroplasty* using oral mucosa grafts in *men and boys* with urethral stricture due to LS causing mechanical problems in voiding or sexual intercourse.	**↑↑**	100% consensus consensus‐based*
We *suggest* meatoplasty in *men and boys* with meatal stenosis due to LS that did not respond to guideline‐conform treatment with, for example, topical corticosteroids.	**↑**	100% consensus consensus‐based*
We *suggest against* dilatation in *men and boys* with urethral or meatal stenosis due to LS, except for palliative purposes.	**↓**	100% consensus consensus‐based*
*Based on the low level of evidence, the guideline development group decided to provide a purely consensus‐based recommendation despite systematic review of evidence (see evidence report).		
Prior to surgery in females, we *recommend* … − that women are informed about and agree to the required topical treatment (usually with corticosteroid ointments, mostly clobetasol propionate 0.05%) before and after surgery; − that women receive interdisciplinary counselling including advice from specialized pelvic floor physiotherapists and sex therapists. For surgical procedures in males, … − we *recommend* histopathological examination of the removed foreskin to confirm LS and to exclude precancerous lesions; − we *recommend* pre‐ and post‐operative treatment with topical corticosteroid ointments (usually clobetasol propionate 0.05%; for example, 4 weeks before and 4–12 weeks after the procedure);	**↑↑**	100% consensus consensus‐based
− we *suggest* removing the complete foreskin by circumcision.	**↑**	

## SPECIFIC FEATURES OF EXTRAGENITAL LS

Compared to genital LS, extragenital manifestation of LS is much less frequent in our latitudes and affects approximately 10%–20% of patients with LS.^9^ It predominantly occurs in women, with a reported female/male ratio of 7:2.^121^ Extragenital LS without concomitant genital disease is very rare. Extragenital LS and morphea share clinical similarities, and intraindividual coexistence of both conditions has been reported.^122^ Clinically, extragenital LS appears as porcelain‐like polygonal papules or plaques (Figure 5). Several morphologic variants have been described for extragenital LS, including bullous, ulcerative, annular, desquamative, telangiectatic, angiokeratomatous, verrucous, and vitiligoid LS.^121^ In bullous extragenital LS, blister formation might be explained by two mechanisms. Firstly, stability of the basal membrane zone is disrupted by interface dermatitis and apoptosis of the basal layer. Secondly, edema of the papillary dermis separates the collagen fibers and flattens the rete ridges.^123^ The majority of lesions are asymptomatic or accompanied by mild itching. In widespread extragenital LS, however, dermal atrophy and sclerosis may cause substantial discomfort. The Koebner phenomenon has been described in the context of LS. Recently, extragenital LS has been reported in association with tattoos.^124^ Extragenital LS can affect the entire body, but predisposed anatomical sites include, in particular, the trunk (sub‐mammary region, abdomen, buttocks, shoulders, wrists, and chest) and proximal extremities.^124^, ^125^ Involvement of the oral mucosa (predominantly lips and buccal area) has been described in very rare cases.^126^, ^127^ However, these cases need to be critically scrutinized, given that oral LP may be misinterpreted as manifestation of LS due to the clinical and histopathological similarities and both diseases may occur simultaneously.

A recent report from the Iraq describes the predominant extragenital manifestation of LS. This raises the question of a different genetic background or different trigger factors.^27^

## FOLLOW‐UP

**Table:** 

We *recommend* regular follow‐up examinations for patients with LS; initially, for example, every 3–6 months and once the disease is stable, for example, every twelve months.	**↑↑**	100% consensus consensus‐based

**Table:** 

We *recommend* that all children with LS are followed by specialists with expertise in treating children with LS.	**↑↑**	100% consensus consensus‐based

**Table:** 

We *recommend* that all adult LS patients who do not respond to topical treatment with potent or ultrapotent corticosteroids or who have precancerous lesions of the vulva or penis are followed by specialists, for example, dermatologists, gynecologists, or urologists.	**↑↑**	100% consensus consensus‐based

**Table:** 

We *recommend* that LS patients with voiding problems are referred to an appropriate specialist.	**↑↑**	100% consensus consensus‐based

**Table:** 

For the follow‐up of patients with LS, we *recommend* the following: − monitoring of treatment effectiveness (including symptom relief/control, normalization of skin color and texture), − asking about problems in voiding, defecation and sexual function, − close monitoring for the development of precancerous or cancerous lesions, − ensuring adherence to treatment/compliance.	**↑↑**	100% consensus consensus‐based

## INTERDISCIPLINARY MANAGEMENT

**Table:** 

We *recommend* referral to other specialists, for example, in the following situations: − if no adequate improvement of clinical signs and/or symptoms after adequate treatment is observed, − if there are complications that require specialized approaches, such as functional impairments requiring surgical treatment or chronic pain syndromes requiring care by a pain specialist, − if psychological support is needed, − if sexological support is needed, − if transition from adolescent to adult medicine is indicated.	**↑↑**	100% consensus consensus‐based

## CONFLICT OF INTEREST STATEMENT

A full list of declared interests is available in the guideline development report (Leitlinienreport) at https://register.awmf.org/de/leitlinien/detail/013‐105
